# Incidence and predictors of postoperative recall of propofol injection pain: a prospective observational cohort pilot study

**DOI:** 10.1016/j.bjao.2026.100537

**Published:** 2026-02-27

**Authors:** Gabriel Bellouni, Matthieu Clanet, Karim Touihri, Amelie Delaporte, Nancy M. Boulos, Kyubin Kim, Tristan Grogan, Brenton Alexander, Sean Coeckelenbergh, Alexandre Joosten

**Affiliations:** 1 Department of Anaesthesiology, CHIREC Hospital, Brussels, Belgium; 2 Department of Anaesthesiology & Perioperative Medicine, David Geffen School of Medicine, University of California Los Angeles, Los Angeles, CA, USA; 3 University of California Los Angeles, Los Angeles, CA, USA; 4 Department of Medicine Statistics Core, David Geffen School of Medicine, University of California Los Angeles, Los Angeles, CA, USA; 5 Department of Anaesthesiology & Perioperative Medicine, University of California San Diego, La Jolla, CA, USA; 6 Department of Anaesthesiology & Perioperative Medicine, University of California Irvine, Irvine, CA, USA; 7 Outcomes Research Consortium, Houston, TX, USA

**Keywords:** anaesthesia induction, injection pain, patient-reported outcomes, postoperative recall, risk factors

## Abstract

**Background:**

Propofol injection pain is common during i.v. induction and is often accompanied by nociceptive behaviours. Whether this pain is subsequently remembered by patients remains poorly characterised. We aimed to estimate the incidence of postoperative day 1 (POD#1) recall of propofol injection pain and to explore unadjusted associations with patient-, procedure-, and technique-related factors in routine clinical practice.

**Methods:**

We conducted a pilot prospective observational cohort study of adults undergoing low- to intermediate-risk noncardiac surgery with i.v. propofol induction between March and August 2025 at a single tertiary centre. The primary outcome was patient-reported recall of propofol injection pain on POD#1, assessed *via* structured interview. Secondary outcomes included recalled pain intensity (0–10 numerical rating scale), comparison with i.v. catheter insertion, and associations with observed nociceptive reactions and injection technique (catheter site, gauge, lidocaine use). All regression analyses were univariate, exploratory, and unadjusted.

**Results:**

Among 1043 patients with POD#1 data, 102 patients (9.8%; 95% confidence interval, 8.1–11.8%) recalled propofol injection pain. Recall was more frequent in women in univariate analyses. Catheter site, gauge, and lidocaine use showed no unadjusted associations with recall. Nociceptive reactions were frequent but did not differentiate patients with and without recall. Among patients with recall, median pain intensity was 7/10 (interquartile range, 5–9), and 99% rated pain as equal to or worse than i.v. cannulation.

**Conclusions:**

Approximately 10% of patients recalled propofol injection pain on POD#1, typically as severe pain. Female sex showed an unadjusted association with recall, whereas injection technique and observed nociceptive reactions did not.

Propofol is the most widely used i.v. hypnotic agent for the i.v. induction of general anaesthesia, yet propofol injection pain remains one of its most frequent and unpleasant adverse effects. Patients typically describe a brief burning or aching sensation beginning within seconds of injection, and anaesthesiologists are familiar with the associated nociceptive behaviours, grimacing, verbal complaint, or hand withdrawal, that accompany it.^1^ Although numerous strategies have been proposed to attenuate propofol injection pain, including lidocaine pretreatment or admixture, venous occlusion techniques, slow injection through a running carrier fluid, attention to catheter size or location, and reformulated emulsions,^1^, ^2^, ^3^, ^4^ the phenomenon persists in routine practice.^5^

Despite the large body of literature addressing methods to *reduce* propofol injection pain, remarkably little is known about whether patients *remember* this pain once awake after surgery. From a patient-centred perspective, postoperative recall may be more relevant than the intraoperative nociceptive event itself. A distressing memory of ‘the injection that burned’ may shape patient satisfaction or contribute to anticipatory anxiety before future procedures. Yet the incidence of recall, and the factors that predispose some patients to remember propofol injection pain while others do not, remains poorly characterised.^5^^,^^6^ Furthermore, it is unclear whether observed behavioural responses reliably predict postoperative memory, or whether simple technique-related factors such as i.v. site, catheter gauge, or lidocaine use meaningfully influence recall.

The primary aim of this pilot prospective observational cohort study was to estimate the incidence of postoperative day (POD) 1 recall of propofol injection pain; secondarily, exploratory analyses examined unadjusted associations between recall and patient-, procedure-, and injection-related factors.

## Methods

This study was approved by the Institutional review board (IRB) of Erasme Hospital in Brussels with the reference number 021/406 on 9 April 2025 (principal investigator: MC). In accordance with IRB requirements, no written consent was requested because the study involved no procedures beyond routine clinical care. Oral consent was documented in the medical record. This was a single-centre prospective observational cohort study conducted at one tertiary hospital in Belgium (Delta hospital in Brussels, Belgium) in compliance with the Declaration of Helsinki and European General Data Protection Regulation (GDPR). Data confidentiality and anonymity were strictly maintained. During the pre-anaesthesia consultation, eligible patients were informed about the study and its objectives; an information sheet was provided. The study was supported solely by departmental funds, with no involvement of any external sponsor. All data were collected at a single tertiary hospital in Belgium. Authors affiliated with US institutions contributed to the study design, statistical analysis, interpretation of the results, and manuscript preparation only. The STROBE checklist has been updated to correspond exactly to the current version of the manuscript, with revised section and page references.

We included all consecutive, French-speaking patients (>18 yr old) scheduled for an elective noncardiac surgery for whom propofol was used for anaesthesia induction. We did not include minors and patients with altered cognition or language barrier that prevented reliable postoperative assessment. Importantly, only French-speaking patients were included to ensure reliable postoperative interviews and assessment of patient-reported recall.

### Intraoperative phase

Anaesthetic management followed standard departmental practice with no additional interventions mandated by the study. During the study period, only a single propofol formulation was in use: propofol 1% (10 mg ml^−1^; B. Braun Medical, Machelen, Belgium) in a long-chain triglyceride lipid emulsion (Intralipid®, Fresenius Kabi, Bad Homburg, Germany). No other concentrations or formulations were available. The following variables were systematically recorded: any use of lidocaine before propofol injection, catheter size (gauge) and insertion site, and anaesthesiologist-observed pain response at injection (yes/no) and type (grimace-hand removal, oral complaint, or other types of nociceptive reaction). Non-pharmacological comfort measures—such as topical anaesthetic agents, cold spray, distraction techniques, tactile ‘gate-control’ stimulation, or structured positive-reinforcement strategies—were not used during cannula insertion or induction, reflecting routine practice. At our institution, dexamethasone is administered after i.v. induction and tracheal intubation, when the patient is unconscious; accordingly, it does not contribute to nociceptive stimuli during propofol injection.

### Postoperative phase

On POD#1, trained study personnel, who were not involved in the intraoperative care and were unaware of the specific hypotheses regarding predictors of recall, conducted structured interviews. Assessors were aware that the study pertained to patient-reported experiences after i.v. induction but were not informed of the specific hypotheses regarding predictors of recall, and they had no access to intraoperative observations or documentation at the time of assessment.

The standardised questionnaire evaluated the presence of **recalled** pain during propofol injection (yes/no), the intensity of the recalled pain if remembered (using the numerical rating scale (NRS) from 0 to 10, and the subjective impact of this experience compared with the catheter insertion placement. Postoperative pain scores (general postoperative pain) were **not collected**, as this was outside the scope of the protocol.

### Primary outcome and variables of interest

The primary outcome was the presence (yes/no) of a patient-reported painful memory of propofol injection on POD#1 using a structured, prompted interview. Patients were asked a standardised question regarding whether they remembered experiencing pain during propofol injection at induction. If recall was reported, pain intensity was quantified using a 0–10 NRS. All assessments were conducted within 24 h after surgery.

Key secondary outcomes included the intensity of the recalled pain (from 0 to 10 on a NRS), the anaesthesiologist’s observation of pain expression at the time of injection (yes/no), and any other explanatory factors (use of lidocaine, catheter gauge and site, age, sex, and selected relevant medical history).

### Sample size estimation

The sample size was determined to provide a precise estimate of the incidence of POD#1 recall of propofol injection pain. Assuming an expected incidence of approximately 10%, a sample size of at least 1000 patients was required to achieve a margin of error of approximately 2% around the incidence estimate.

### Statistical analysis

Patient characteristics were summarised overall and stratified by recall status (yes *vs* no). Continuous variables were reported as mean (sd) or median with interquartile range (IQR), depending on distribution, and compared using Student’s *t*-tests. Categorical variables were reported as counts and percentages and compared using χ^2^ tests or Fisher’s exact tests where expected cell counts were less than 5.

The primary outcome, incidence of recalled propofol injection pain on POD#1, was estimated with a binomial 95% confidence interval (Wilson method). Univariate logistic regression was used to explore associations between patient-, procedure-, and technique-related variables and recall. Results were expressed as odds ratios (ORs) with 95% confidence intervals (CIs) and two-sided *P*-values. All regression analyses were univariate, no multivariable adjustment was performed, and the results should therefore be interpreted as exploratory and hypothesis-generating. Missing data were handled using complete-case analysis; patients without POD#1 interview data were excluded from all analyses, and no imputation was performed. Fifty-seven patients who could not be reached for the POD#1 interview were excluded from all analyses. For predictor variables, missingness was 0%, and no imputation was performed. All statistical tests were two sided, and *P*<0.05 was considered statistically significant. *P*-values for baseline characteristics are reported descriptively to facilitate comparison between groups and should not be interpreted as evidence of causal associations. Analyses were conducted using R version 4.4.3 (R Foundation for Statistical Computing, Vienna, Austria).

## Results

**Fig 1:**
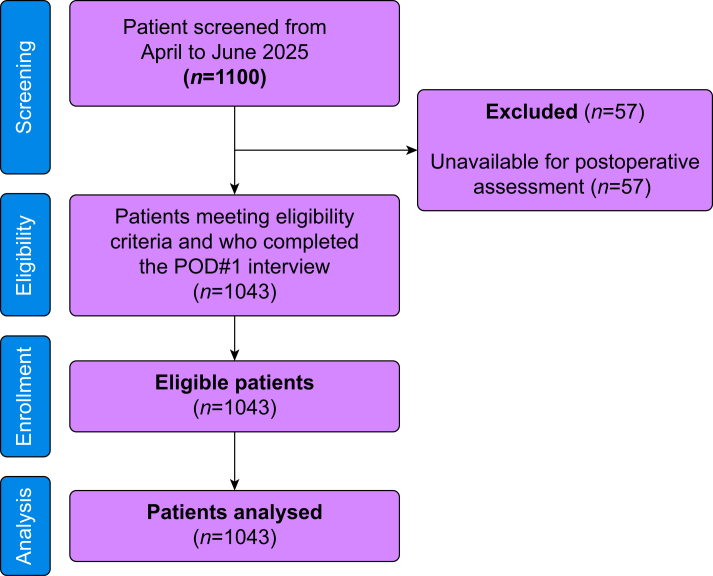
Flow chart. POD#1, postoperative day 1.

Between April and August 2025, 1100 consecutive patients were screened and all met eligibility criteria. Of these, 1043 patients (94.8%) completed the POD#1 interview and were included in the analysis. Fifty-seven patients (5.2%) did not answer the postoperative phone call despite multiple attempts, and their data could not be analysed. These patients were lost to follow-up after routine postoperative discharge; no withdrawals, protocol deviations, or adverse events accounted for the missing data. A flow diagram is provided in Figure 1.

The median (IQR) age of the cohort was 50 (18–90) yr, and 49% were women. Baseline patient and clinical characteristics are summarised in Table 1. In univariate analyses, female sex showed an unadjusted association with recall. Catheter gauge, insertion site, and use of lidocaine during induction did not significantly differ between groups. Only 4% of patients received a preoperative benzodiazepine.

**Table 1:** Baseline characteristics of the patients. Values are given as *n* (%) or median (interquartile range). Categorical variables compared using χ^2^ tests; continuous variables using Student’s *t*-tests. Bold values represnt significant p value ( *P* < 0.05). COPD, chronic obstructive pulmonary disease; NRS, numerical rating scale; POD, postoperative day.

Variables	Overall	Recall pain at POD#1		
		No	Yes	*P*-value
	(*n*=1043)	(*n*=941)	(*n*=102)	
Sex, female	513 (49)	449 (48)	64 (63)	**0.004**
Age (yr)	50 (35–69)	51 (36–69)	44 (33–60)	**0.020**
Height (cm)	171.8 (9.9)	171.8 (9.9)	171.3 (10.2)	0.622
Weight (kg)	82.3 (18.4)	82.3 (18.1)	82.2 (20.7)	0.945
BMI (kg m^−2^)	28 (24–32)	28 (24–32)	27 (22–34)	0.900
ASA 2	585 (56)	526 (56)	59 (58)	0.707
ASA 3	458 (44)	415 (44)	43 (42)	0.707
Size of catheter				
18 Gauge	396 (38)	359 (38)	37 (36)	0.711
20 Gauge	450 (43)	400 (43)	50 (49)	
22 Gauge	197 (19)	182 (19)	15 (15)	0.256
Position of catheter				0.981
Forearm	367 (35)	331 (35)	36 (35)	
Arm	18 (1.7)	17 (1.8)	1 (1.0)	>0.999
Hand	658 (63)	593 (63)	65 (64)	0.888
Use of lidocaine	581 (56)	525 (56)	56 (55)	0.864
Midazolam before surgery	37 (4)	33 (4)	4 (4)	0.777
Chronic pain	84 (8)	78 (8)	6 (6)	0.396
Asthma	173 (17)	158 (17)	15 (15)	0.591
COPD	44 (4)	36 (4)	8 (8)	0.067
Coronary artery disease	47 (5)	45 (5)	2 (2)	0.310
Diabetes (all types)	320 (31)	286 (30)	34 (33)	0.541
Depression	36 (4)	33 (4)	3 (3)	>0.999
Hypertension	261 (25)	238 (25)	23 (23)	0.543
Hyperthyroidie	7 (0.7)	6 (0.6)	1 (1.0)	0.515
Hypothyroidie	16 (2)	13 (1)	3 (3)	0.200
Chronic kidney disease	24 (2)	19 (2)	5 (5)	0.077
Obesity	417 (40)	380 (40.4)	37 (36.3)	0.421

On POD#1, 102 patients (9.8%; 95% CI, 8.1–11.8%) reported a memory of propofol injection pain. Among patients with recall, the median intensity of the remembered pain was high (median NRS 7, [IQR, 5–9]). Nearly all recall-positive patients (99%) rated propofol injection pain as equal to or more severe than their i.v. catheter insertion (≈51% equal, 48% worse; *P*<0.001) (Table 2).

**Table 2:** Secondary outcomes. Values are given as *n* (%) or median (interquartile range). Categorical variables compared using χ^2^ tests; continuous variables using Student’s *t*-tests. NRS, numerical rating scale; POD, postoperative day.

Variables	Overall	Recall pain at injection		
		No	Yes	*P*-value
	(*n*=1043)	(*n*=941)	(*n*=102)	
Sensation during propofol injection				
Grimace	122 (12)	112 (12)	10 (10)	0.531
Verbal complain	130 (13)	116 (12)	14 (14)	0.685
Hand withdrawal	123 (12)	110 (12)	13 (13)	0.754
Other reaction	301 (29)	269 (29)	32 (31)	0.555
Any reaction	676 (65)	607 (65)	69 (68)	0.528
Recall pain at POD#1				
Intensity of pain (NRS)	0 (0–0)	0 (0–0)	7 (5–9)	<0.001

**Fig 2:**
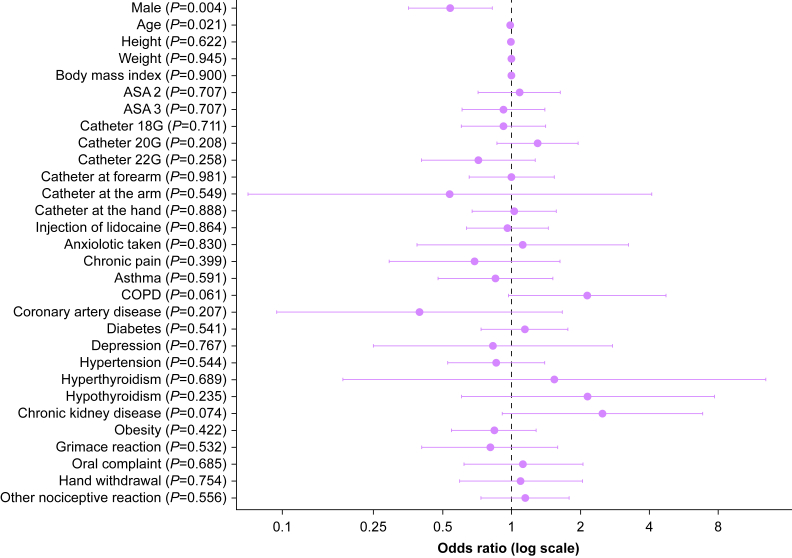
Univariate association between different variables and propofol injection pain. COPD, chronic obstructive pulmonary disease.

Observed nociceptive behaviours during injection were common in both groups but did not meaningfully discriminate recall (68% among those with recall *vs* 65% among those without recall; *P*=0.528). In exploratory univariate analyses, catheter site, catheter gauge, and use of lidocaine were not significantly associated with recall. Detailed univariate results are presented in Table 3. Figure 2 shows univariate associations between different variables and propofol injection pain.

**Table 3:** Exploratory univariable associations with recall of propofol injection pain on postoperative day 1 with OR and 95% CI. All associations are based on univariate, unadjusted analyses and should be interpreted as exploratory. Bold values represnt significant p value ( *P* < 0.05). CI, confidence interval; COPD, chronic obstructive pulmonary disease; OR, odds ratio.

Variable	OR (95% CI)	*P*-value
Male	0.54 (0.36–0.83)	**0.004**
Age	0.99 (0.98–1.00)	0.021
Height	1.00 (0.97–1.02)	0.622
Weight	1.00 (0.99–1.01)	0.945
Body mass index	1.00 (0.97–1.04)	0.900
ASA 2	1.08 (0.72–1.64)	0.707
ASA 3	0.92 (0.61–1.40)	0.707
Catheter 18G	0.92 (0.60–1.41)	0.711
Catheter 20G	1.30 (0.86–1.96)	0.208
Catheter 22G	0.72 (0.41–1.27)	0.258
Catheter at forearm	1.01 (0.66–1.54)	0.981
Catheter at the arm	0.54 (0.07–4.09)	0.549
Catheter at the hand	1.03 (0.67–1.58)	0.888
Injection of lidocaine	0.97 (0.64–1.45)	0.864
Anxiolotic taken	1.12 (0.39–3.24)	0.830
Chronic pain	0.69 (0.29–1.63)	0.399
Asthma	0.85 (0.48–1.52)	0.591
COPD	2.14 (0.97–4.74)	0.061
Coronary artery disease	0.40 (0.10–1.67)	0.207
Diabetes (diabetes mellitus and diabetes insipidus)	1.15 (0.74–1.77)	0.541
Depression	0.83 (0.25–2.77)	0.767
Hypertension	0.86 (0.53–1.40)	0.544
Hyperthyroidia	1.54 (0.18–12.94)	0.689
Hypothyroidia	2.16 (0.61–7.72)	0.235
Chronic kidney disease	2.50 (0.91–6.85)	0.074
Obesity	0.84 (0.55–1.28)	0.422
Reaction observed at induction		
Grimace	0.81 (0.41–1.59)	0.532
Oral complain	1.13 (0.62–2.05)	0.685
Hand withdrawal	1.10 (0.60–2.04)	0.754
Other	1.14 (0.73–1.78)	0.556

## Discussion

In this prospective observational pilot cohort study, ∼10% of patients recalled propofol injection pain on the day after anaesthesia. Although nociceptive behaviours during induction were frequent, they did not reliably predict postoperative recall, highlighting a dissociation between immediate behavioural responses and subsequent memory of pain. In univariate, exploratory analyses, female sex showed an unadjusted association with recall, whereas injection-related technical factors and observed nociceptive reactions did not. The possible association between female sex with recall echoes previous reports of sex-related differences in pain perception and memory consolidation.^6^ Women have been shown to have greater sensitivity to experimental pain stimuli, which may reflect hormonal modulation of nociceptive processing and heightened limbic activation during noxious events. When recall occurred, patients generally described the pain as intense, with a median NRS score of 7, and almost all judged it to be equal to or more severe than pain from i.v. cannulation. These findings highlight postoperative recall of propofol injection pain as a distinct and clinically relevant patient-centred outcome.

Our observation that only a minority of patients recall propofol injection pain, despite the high frequency of observed nociceptive reactions, underscores the dissociation between behavioural responses and memory formation. Anaesthesiologists commonly interpret grimacing, verbalisation, or hand withdrawal as indicators of patient experience, yet these rapid, reflexive behaviours do not necessarily reflect what is encoded into memory. Several mechanisms may explain this disconnect. Memory consolidation occurs within a narrow window of conscious awareness just before loss of responsiveness; therefore, patients may display nociceptive behaviours but lose consciousness too quickly for the stimulus to be encoded. Conversely, the small proportion of patients who recalled pain despite no observable reaction may include individuals who are less outwardly expressive (‘stoic’) or whose psychological factors, such as anxiety, attentional focus, or emotional salience, facilitated memory formation despite minimal behavioural manifestation. Importantly, patients were not primed about the possibility of pain or recall, and postoperative interviewers were blinded to intraoperative observations. Together, these findings suggest that propofol injection pain recall is influenced not only by nociceptive intensity but also by individual differences in psychological processing and memory encoding.

From a patient perspective, the memory of ‘the injection that burned’ may be more consequential than the brief nociceptive event itself. Although only 10% recalled propofol injection pain, those who did described severe pain, raising the possibility that this memory may shape their overall impression of anaesthesia care. Previous literature suggests that expectations, anxiety, and cognitive framing strongly influence satisfaction; thus, the clinical relevance of propofol injection pain may depend more on whether it becomes a salient postoperative memory than on the transient intraoperative stimulus alone. Future research incorporating patient satisfaction, expectations, and communication strategies will be important to clarify this.

The dissociation between nociceptive behaviours and recall warrants further consideration. Although two-thirds of patients displayed some behavioural response (e.g. grimacing, hand withdrawal), the vast majority of these individuals did not subsequently recall pain. One explanation is that the behavioural response reflects a rapid spinal or subcortical nociceptive reflex, whereas memory encoding requires a brief but critical window of conscious awareness before loss of consciousness. Depending on injection speed and the timing of induction agents, many patients may exhibit a reflexive response but lose consciousness before the event is fully encoded.

Conversely, a small subset of patients (*n*=35) reported recall despite no observable reaction. These cases may represent individuals who are less outwardly expressive (‘stoic’) yet still experience pain, or patients whose attentional focus or emotional salience was higher, thereby facilitating memory encoding despite minimal behavioural manifestation. Importantly, patients were not primed by investigators regarding the possibility of pain or recall; no anticipatory suggestions were provided. Thus, priming cannot account for these findings. Together, these observations suggest that propofol injection pain recall depends not only on nociceptive intensity but also on individual differences in attention, anxiety, emotional salience, and the timing of memory consolidation relative to loss of consciousness. These factors may explain why behavioural responses alone are poor predictors of postoperative recall. Lastly, an important clinical implication of these findings relates to the scope of preventive strategies. Although propofol injection pain is frequent at induction, only a minority of patients (∼10%) recalled this pain on POD#1. This raises the question of whether universal mitigation strategies aimed at eliminating all peri-induction pain are necessary, or whether preventive efforts should be more selectively targeted. Although complete avoidance of pain remains a general principle of anaesthesia care, our findings suggest that postoperative recall, rather than transient nociceptive responses alone, may represent a more patient-relevant outcome. Future studies should thus aim to identify patients at higher risk of recall and evaluate whether targeted preventive strategies improve patient-centred outcomes more efficiently than universal approaches.

In the future, the ideal study design would be to rigorously evaluate propofol injection pain and its postoperative recall would require a fully standardised induction protocol. This would include controlling the speed of propofol administration, the rate of the carrier fluid, the formulation of propofol, the exact timing and dose of lidocaine pretreatment (with and without venous occlusion), and the choice and location of the i.v. catheter. Such a study should use a randomised design comparing well-defined mitigation strategies (e.g. lidocaine pretreatment, admixture techniques, alternative formulations, or adjunctive agents) *vs* standardised control conditions. In addition to capturing intraoperative nociceptive behaviours, it should incorporate objective physiological markers of nociceptive processing (e.g. EEG-derived indices or nociception monitors) to better understand the temporal relationship between nociceptive input and memory formation. Postoperative assessments should occur at multiple time points to characterise memory consolidation and decay. Finally, the study should be adequately powered to detect clinically meaningful differences in both immediate pain and postoperative recall, use multivariable modelling to account for confounders, and recruit a diverse population across multiple centres to ensure generalisability. Such a design would provide the most definitive evidence regarding the mechanisms, predictors, and preventable components of propofol injection pain.

Our study has several strengths. We prospectively enrolled more than 1000 consecutive patients over 5 months, providing one of the largest datasets on propofol injection pain recall to date. Recall was assessed systematically on POD#1, reducing bias from variable documentation or delayed patient contact. The large sample size allowed us to estimate the incidence with high precision and explore multiple patient- and technique-level predictors. Nevertheless, certain limitations merit consideration. First, this was a single-centre European study, which may limit generalisability to settings with different propofol formulations, anaesthesia protocols, or i.v. access practices. Second, several procedural variables known to influence propofol injection pain, such as injection speed, carrier-fluid rate, and the timing and dose of lidocaine, were not standardised, reflecting routine practice but introducing uncontrolled variability. Variability in these parameters may have introduced uncontrolled heterogeneity and could have attenuated or obscured associations between technique-related factors and postoperative recall. Third, all analyses were univariate and unadjusted; therefore, residual confounding cannot be excluded and findings should be interpreted as exploratory. Fourth, recall was assessed only on POD#1, which may have resulted in misclassification. Some patients may have experienced early memory extinction leading to false-negative recall, whereas others may have reconstructed or reinterpreted peri-induction experiences after surgery, potentially resulting in false-positive recall. Longitudinal assessments at multiple postoperative time points would better help characterise the trajectory of recall and its influence on longer-term patient satisfaction and perioperative anxiety. Fifth, we did not assess patient satisfaction, which is an important limitation. Satisfaction often reflects a broader appraisal of perioperative care and does not necessarily correlate with pain intensity alone. Previous research suggests that reasonable patients frequently anticipate some degree of procedural discomfort, and that expectations strongly shape satisfaction—sometimes more than pain scores themselves. It is conceivable that similar mechanisms apply to propofol injection pain: some patients may consider it trivial or acceptable within the context of surgery, particularly if they are informed in advance, whereas others may perceive it as disproportionately distressing. Because we did not evaluate satisfaction or expectation-related factors, we cannot determine how recalled propofol injection pain influenced overall patient experience. Future studies should incorporate measures of satisfaction, expectations, and communication to better understand the clinical relevance of propofol injection pain recall. Sixth, inclusion was restricted to French-speaking patients, which may introduce selection bias and limit generalisability to more diverse populations. Language proficiency and cultural context may influence pain expression, recall, and the communication of subjective experiences, and the present findings may not be fully generalisable to more linguistically and culturally diverse populations. Additionally, individuals lost to follow-up may differ systematically from those reached on POD#1, although the proportion was small (5.2%). These factors should be considered when interpreting the external validity of our findings. Finally, because this was an exploratory pilot study, multiple hypothesis testing correction was not applied. As a result, some associations—particularly those with *P*-values near the significance threshold—may reflect chance findings. These exploratory results should therefore be interpreted cautiously and validated in larger confirmatory studies. Moreover, because all analyses were univariate and unadjusted, these findings should be considered exploratory and hypothesis-generating only.

### Conclusions

Approximately 10% of patients recall propofol injection pain on POD#1, typically as severe pain. In this exploratory pilot cohort study, female sex showed an unadjusted association with recall, whereas catheter characteristics, lidocaine use, and observed nociceptive reactions did not show unadjusted associations. These findings highlight postoperative recall of propofol injection pain as an understudied patient-centred outcome and suggest a disconnect between nociceptive responses and memory formation. Future studies should clarify the mechanisms linking nociceptive input, psychological factors, and memory encoding, and determine whether interventions aimed at attenuating propofol injection pain meaningfully improve postoperative experience or should instead be selectively targeted.

## Authors’ contributions

Study design/conception: all authors

Study conduct: GB, MC, KT

Data analysis: TG, AJ

Writing paper: AJ, GB, AD

Editing paper: all authors

Revising paper: all authors

## Data availability statement

The data supporting the findings of this study are not publicly available owing to institutional and regulatory restrictions but are available from the corresponding author upon reasonable request.

## Funding

Departmental funding.

## Declarations of interest

The authors declare that they have no conflicts of interest.
